# *Sylphella puccoon* gen. n., sp. n. and two additional new species of aquatic oligochaetes (Lumbriculidae, Clitellata) from poorly-known lotic habitats in North Carolina (USA)

**DOI:** 10.3897/zookeys.451.7304

**Published:** 2014-11-03

**Authors:** Pilar Rodriguez, Steven V. Fend, David R. Lenat

**Affiliations:** 1 Department of Zoology and Animal Cell Biology, Faculty of Science and Technology, University of the Basque Country, Box 644, 48080 Bilbao, Spain; 2 U.S. Geological Survey, 345 Middlefield Rd., Menlo Park CA 94025, USA; 3 Lenat Consulting, 3607 Corbin Street, Raleigh NC 27612, USA

**Keywords:** Lumbriculids, biodiversity, acidic waters, pocosin soils, North America

## Abstract

Three new species of Lumbriculidae were collected from floodplain seeps and small streams in southeastern North America. Some of these habitats are naturally acidic. *Sylphella puccoon* **gen. n.**, **sp. n.** has prosoporous male ducts in X–XI, and spermathecae in XII–XIII. Muscular, spherical atrial ampullae and acuminate penial sheaths distinguish this monotypic new genus from other lumbriculid genera having similar arrangements of reproductive organs. *Cookidrilus pocosinus* **sp. n.** resembles its two subterranean, Palearctic congeners in the arrangement of reproductive organs, but is easily distinguished by the position of the spermathecal pores in front of the chaetae in X–XIII. *Stylodrilus coreyi* **sp. n.** differs from congeners having simple-pointed chaetae and elongate atria primarily by the structure of the male duct and the large clusters of prostate cells. Streams and wetlands of Southeastern USA have a remarkably high diversity of endemic lumbriculids, and these poorly-known invertebrates should be considered in conservation efforts.

## Introduction

In contrast to larger streams and rivers, aquatic habitats such as wetlands, small headwater tributaries or springs have received little attention. As these habitats are not directly connected to each other by flow, and their physical structure can vary greatly from one location to another, they may be expected to support a distinct invertebrate fauna, demonstrate greater variation in taxonomic composition, and have patterns of taxa richness and assemblage variation that do not correlate with adjacent main stream habitats. Riverine wetlands studies in North America indicate high macroinvertebrate taxonomic richness, and greater assemblage variation compared to nearby riffle communities (Curry et al. 2012).

Biological assessment of swamp streams in the southeastern USA has been particularly difficult, especially in naturally acidic areas, since the unusual conditions result in a distinctive fauna with relatively low diversity and abundance of taxa commonly used to indicate excellent water quality (e.g., Ephemeroptera, Plecoptera, Trichoptera – EPT). The North Carolina Division of Environmental Management has struggled with ratings for swamp streams in North Carolina for many years, and for some regions of the state there is still insufficient information to assign water quality ratings (NCDENR 2013). Separate criteria (Lenat 2003) have been established for some swamp streams, taking into account their much lower EPT and total taxa richness relative to more typical streams. Documenting the presence of rare or endemic taxa is one way to address the requirement for conservation of these habitats. A reasonable assessment of such habitats will only be possible with a better understanding of their little-known fauna.

The southeastern region of North America has revealed a great degree of endemicity in the oligochaete family Lumbriculidae. Recent collections from the Sandhills and Coastal Plain ecoregions of North Carolina have resulted in the description of new, and probably endemic, lumbriculid species and genera (Fend and Lenat 2007, 2012). The objective of present study is to contribute to the knowledge of the oligochaete fauna inhabiting poorly known aquatic habitats in that region, with the description of three new lumbriculid species, one of which is assigned to a new genus. Knowledge of the communities of these insufficiently studied habitats is essential to undertake any conservation plan of biodiversity in fluvial catchments.

## Material and methods

Oligochaetes were usually collected by disturbing the substrate and then sweeping through this area with a 300 μm mesh net. Samples were elutriated to remove the heavier sediments. Most collections were live-picked at the sample site, but some material was fixed whole in 10% buffered formalin and brought back to the laboratory for sorting. Field-picked specimens were relaxed by the addition of small amounts of alcohol, and then they were fixed in formalin and/or Bouin’s solution. Formalin-preserved specimens were transferred to 70% alcohol after one day, for long term storage.

Most worms were whole-mounted or longitudinally dissected, stained with Harris’ hematoxylin or borax carmine, dehydrated through an alcohol series, transferred to methyl salicylate and slide mounted in Canada balsam. A few specimens were sagitally sectioned at 7 μm, slide mounted, and stained in hematoxylin and eosin Y.

Drawings of the reproductive system and chaetae were made using a camera lucida. In the descriptions of the male duct and spermatheca, the term *ental* indicates a position that is inner or deep within the body, as opposed to *ectal* for an outer or near-surface position. In the descriptions of chaetae, *proximal* is used to describe a position near the symmetry axis of the body, as opposed to *distal*. Segment numbers are shown in Roman numerals; intersegments are given as Arabic numerals; e.g., “9/10” to represent the intersegment of IX and X. Holotype and paratype specimens are deposited in the U.S. National Museum of Natural History, Smithsonian Institution (USNM), Washington D.C., USA; California Academy of Sciences (CASIZ), San Francisco, California, USA; and Museo Nacional de Ciencias Naturales (MNCN), Madrid, Spain. Non-type specimens are in the authors’ collections.

### Abbreviations in the figures

a: atrium, aa: atrial ampulla, ad: atrial duct, ae: atrial epithelium, am: atrial muscle layer, b: brain, bv: blood vessel, cc: chloragogen cells, cg: chaetal gland, ch: chaeta, dv: dorsal blood vessel, e: efferent duct of nephridium, ff: female funnel, fp: female pore, g: gut, i: intestine, mp: male pore, o: ovary, oc: oocytes, pg: pharyngeal glands, ph: pharynx, pr: prostate, prj: prostate junction, p: penis, ps: penis cuticular sheath, pt: prostomium, sa: spermathecal ampulla, sd: spermathecal duct, sf: sperm funnel, siv: supraintestinal blood vessel, sp: spermatheca, spp: spermathecal pores, t: testis, vd: vas deferens.

### Study area

The new species were collected at five North Carolina locations: one site in the central Piedmont region (UT Pokeberry Creek), two sites in the eastern Coastal Plain (Pettiford Creek and Lake Run) and two sites in the Sandhills (Drowning Creek and Anderson Creek). The Sandhills area is located between the Piedmont and Coastal Plain regions, in the southeastern part of North Carolina. The Coastal Plain and Sandhills sites are humic (“brown-water”) systems. Coordinates for sampling sites are given in WGS84.

The unnamed tributary (UT) to Pokeberry Creek was sampled near the town of Pittsboro in Chatham County, N35.8267, W79.1013. This is part of a floodplain complex of seeps and pools that have surface water only during fall, winter and spring months. The clay soils in the Pokeberry Creek catchment produce small streams (seeps) with a limited hyporheic zone and reduced groundwater storage, causing them to go dry during summer months. These shallow seeps are about 0.5 m wide, with a substrate of clay and decomposing leaves. They originate at the base of a steep hill, largely fed by groundwater, and flow for about 200 m into Pokeberry Creek, within a totally forested area. There were no water chemistry samples from the Pokeberry seeps, but samples from nearby streams suggest pH values close to 7.

Pettiford Creek drains a pocosin area (nutrient-poor, forested or shrub wetland) of the Croatan National Forest in Carteret County. The sampling site (N34.7471, W77.0221) was both upstream and downstream of Forest Service Road 128, also known as Millis Road. Pettiford Creek is about 5 m wide in constricted areas (bridges), but has a much wider braided channel elsewhere (>100 m). The substrate is mostly detritus over a fine sand base. This stream has been frequently used as a reference location by the North Carolina Division of Water Quality. Water pH values from this stream were 3.6 in 2004 and 3.4 in 2010, and conductivity was low (50–85 μS/cm) (NCDENR 2005, 2011). The dominant invertebrates were isopods, amphipods and chironomids, as expected for a swamp stream in this geographic area, but EPT taxa richness was higher than expected for such a low pH stream, with about 10 species per collection (NCDENR 2011). In addition to the new species described here, the lumbriculid fauna in Pettiford Creek is relatively rich, including *Rhynchelmis croatanensis* Fend & Lenat (type locality), *Martinidrilus arenosus* Fend & Lenat, *Altmanella lenati* Fend (type locality), *Eclipidrilus lacustris* (Verrill), *Eclipidrilus breviatriatus* Fend & Lenat, *Eclipidrilus microthecus* Fend & Lenat, and Eclipidrilus cf. fontanus Wassell (Fend 2009, Fend and Lenat 2007, 2010, 2012).

Lake Run drains Little Singletary Lake in Bladen County; samples were collected at State Road (SR) 1325, N34.7773, W78.6646. This stream was sampled for benthic macroinvertebrates in 1981, as part of a study of naturally acidic streams (Lenat, unpublished). At that time, the pH was found to be consistently less than 3.8, with a substrate of fine sand and clay overlain by leaves and woody debris. Stream width was 2–4 m, with a maximum depth of 1.2 m. Conductivity was low, with a range of 45–56 μS/cm. Lake Run also supported 11 EPT species usually considered to be intolerant (Lenat, unpublished data from 1981). In addition to the *Cookidrilus* species described here, the present collection included the lumbriculids *Altmanella lenati* and *Martinidrilus arenosus*.

Drowning Creek was sampled at SR 1004 on the Richmond County/Moore County border, N35.0662, W79.5496. A NCDWQ collection in July 2006 recorded a pH of 5.6, and conductivity was 26 μS/cm in the main channel. This site is about 2.5 km upstream of a reach classified as Outstanding Resource Water (NCDENR 2007), which received an Excellent classification based on high EPT taxa richness (29–30) and low NC Biotic Index values (≤ 4.5). However, the collections cited in this paper are limited to floodplain seeps and pools. Seeps were usually less than 2 m wide, although often forming a braided channel; the substrate was fine sand and detritus, sometimes with patches of aquatic plants or filamentous algae. Water quality in the floodplain is assumed to be similar to that of the main channel of Drowning Creek, which supports a diverse hyporheic oligochaete fauna, with 15 lumbriculid species known from a small (about 200 m) stream segment (Lenat and Fend, unpublished). In addition to the new *Cookidrilus* and *Stylodrilus* species described herein, lumbriculids collected from these seasonally inundated habitats included *Altmanella lenati*, Eclipidrilus cf. fontanus, *Martinidrilus carolinensis* Fend & Lenat and an as-yet undescribed athecate species.

Anderson Creek is a small tributary to the Lower Little River at SR 2031 in Harnett County, N35.2661, W78.8192. Based on earlier studies, conductivity is low (49 μS /cm) and pH slightly acidic (5.0–5.9); the stream has a sand-gravel substrate and is classified as “Good” based on a moderate EPT species richness (NCDENR 2004). Lumbriculid collections from this site included a single specimen of the new *Cookidrilus* species, *Altmanella lenati*, Eclipidrilus cf. fontanus, *Martinidrilus carolinensis*, and at least three undescribed species.

## Results

### Sylphella gen. n.

Taxon classificationAnimaliaLumbriculidaLumbriculidae

http://zoobank.org/06570C13-DB67-418B-B1CC-EC2815B80DFF

#### Diagnosis of the genus *Sylphella* gen. n.

Simple-pointed chaetae. Two pairs of testes and one pair of ovaries. Ovaries in first segment behind testes. Male pores paired in X and XI, female pores paired in the intersegment 12/13. Male ducts prosoporous. Atrial duct forms a penis within a penial sac, distally covered by a cuticular sheath. Two pairs of spermathecae, beginning in the ovarian segment.

#### Type species.

*Sylphella puccoon* sp. n.

### Sylphella puccoon sp. n.

Taxon classificationAnimaliaLumbriculidaLumbriculidae

http://zoobank.org/3D6F9393-7C28-4FBF-A362-37A042364047

Figs 1 , 2 , 3

#### Holotype.

USNM 1251692: a dissected worm, stained in Harris’ hematoxylin, mounted in Canada balsam (collected 23 Jan 2009).

#### Paratypes

all from the type locality. USNM 1251693-1251698: 7 Jan 2009, 1 whole mount; 23 Jan 2009, 3 dissected; 30 Jan 2009, 2 sectioned (1 sagittal, 1 transverse). MNCN 16.03/3083: 14 Jan 2009, 2 dissected. CASIZ 197898: 23 Jan 2009, 3 dissected.

#### Type locality.

An unnamed, very small tributary (seep) to Pokeberry Creek, Chatham Co., North Carolina, USA.

#### Etymology.

The genus name refers to *Sylph*, the Latin name of an elemental spirit of the air that suggests the Latin *silva*, for woodland, followed by the Latin diminutive -*ella*. The specific name *puccoon* is the Algonquian Indian word which means pokeberry (*Phylolacca* species).

#### Other material.

7 Jan 2009, 2 whole mounts. 14 Jan 2009, 3 dissected and 3 whole mounts; 11 in alcohol. 23 Jan 2009, 9 dissected and 1 whole mount; 3 in alcohol. 30 Jan 2009, 2 sectioned for histological study. All specimens (including the type series) collected by D.R. Lenat from the type locality.

#### Description

**(based on mated specimens).** Number of segments 65–83. Length of fixed worms 15–25 mm. Diameter of the body from 14 unmounted worms in lateral aspect (measured to 0.01 mm): 0.44–0.66 mm in VIII (mean 0.51 mm), 0.45–0.68 mm at clitellum (mean 0.54 mm), and 0.50–0.76 mm (mean 0.61 mm) at mid-body.

**Figure 1.**
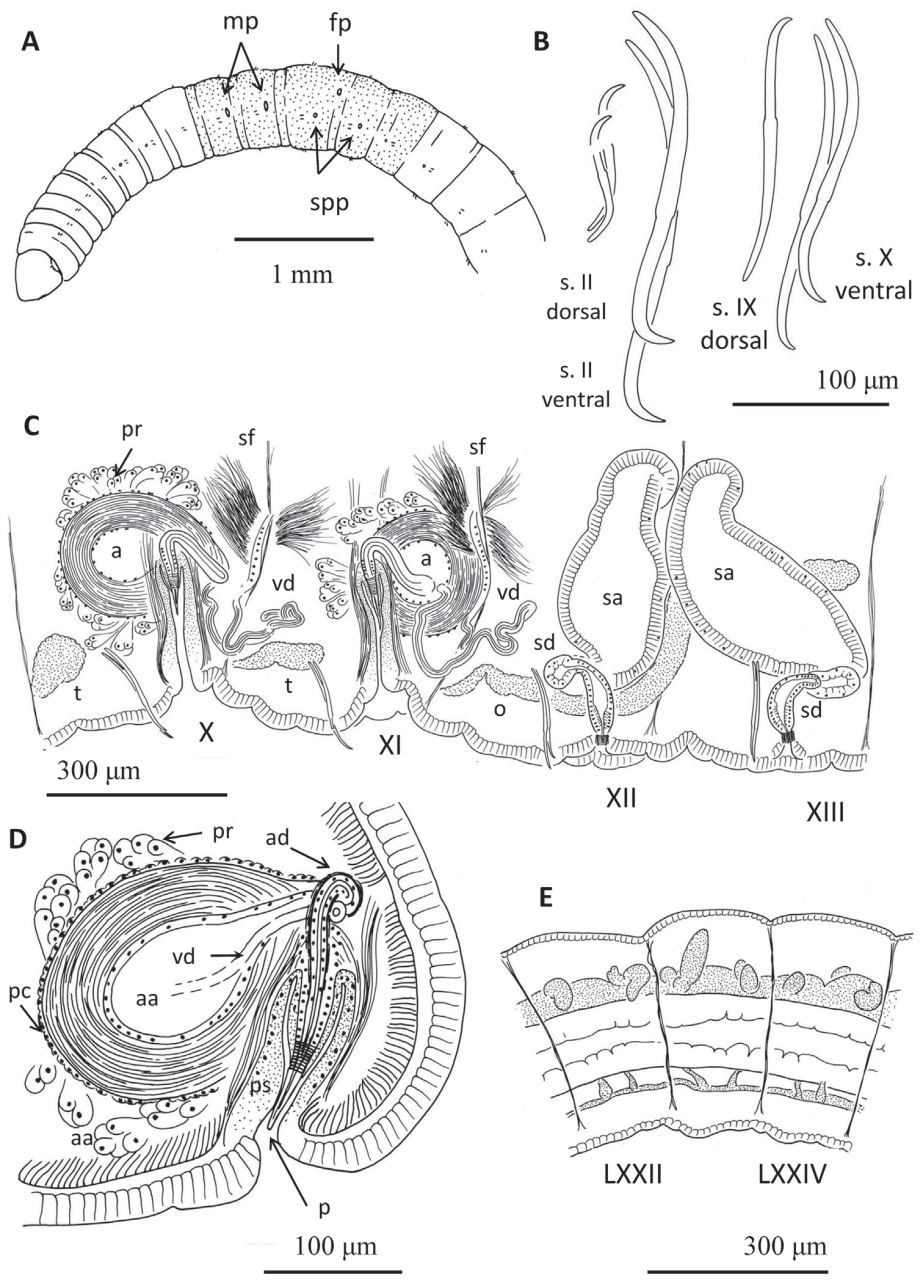
Drawings of *Sylphella puccoon* gen. n., sp. n. **A** Anterior part of the body showing secondary annulations, clitellum and position of genital pores **B** chaetae of segment II and clitellar region **C** schematic drawing of reproductive organs (female funnel obscured by ovary) **D** detail of atrium **E** posterior lateral blood vessels.

**Figure 2.**
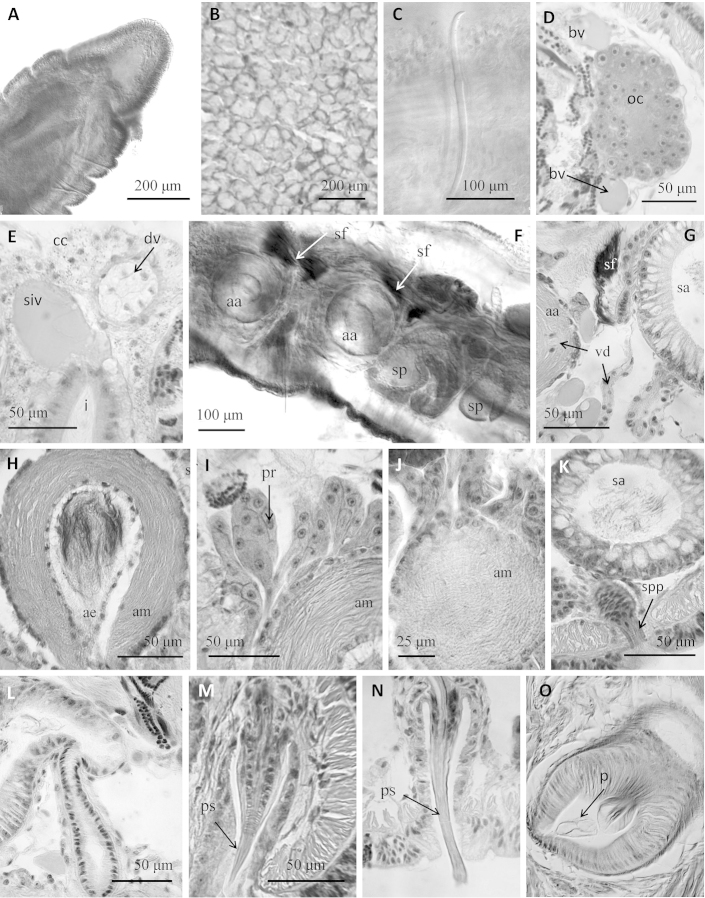
*Sylphella puccoon* gen. n., sp. n. **A** Anterior part of the worm, showing prostomium **B** clitellar epidermis **C** chaeta in XII **D** egg sac containing oocytes and some blood vessels **E** dorsal vessel showing the cardiac cells and supra-intestinal vessel, dorsal to the intestine in segment XVII **F** reproductive segments, showing two atria, with their respective sperm funnels, and spermathecae of an unmated specimen **G** sperm funnel on the septum behind the atrium **H** atrial ampulla with sperm in the lumen, showing the several layers of musculature **I** prostatic cells forming small clusters over the atrial ampulla **J** cross-hatched muscular fibers shown at the surface of the atrial ampulla **K** Spermathecal ampulla with loose sperm in the lumen **L** spermathecal duct **M** penis within the penial sac, with conical penial sheath. For comparison **N** penis with tubular cuticular sheath in *Styloscolex japonicus*, and **O** penis with a soft cuticular layer in *Lumbriculus japonicus*. **D, E, G–O** histological sections of reproductive organs, other photographs from stained whole mounts or dissected specimens.

Prostomium rounded-conical, 270–400 μm long, width about the same as length. Secondary annulation (narrow ring in anterior part of segment) from segment V; present but weak in post-clitellar segments (Fig. 1A). Epidermis 10–15 μm high in anterior segments. Clitellum annular, from segment X to XIV, with epithelium up to 25–35 μm high, formed by unordered, glandular cells (Fig. 2B). Longitudinal muscles up to 32 μm thick in anterior segments. Chaetae sigmoid, simple-pointed with strongly curved distal tip; ventral chaetae larger than corresponding dorsal chaetae in anterior segments (Table 1). Ventrals largest in II to about XIII (126–204 μm long, up to 7 μm thick), anterior dorsals distinctly smaller and thinner, (60–130 μm long, 4 μm thick) (Fig. 1B); maximum ventral chaeta length about 1.6 that of dorsals in preclitellar segments. Ventral chaetae only slightly larger than dorsals in post-citellar segments. Nodulus at about 0.32–0.46 (mean = 0.40) from the distal end.

**Table 1.** Length (μm) of chaetae in *Sylphella puccoon* gen. n., sp. n. (measurements on one whole-mounted specimen from Pokeberry Cr., North Carolina, USA 14 Jan 2009).

Segment	II	III	IV	V	VI	VII	VIII	IX	X	Posterior
**Dorsal**	63	–	88	90	103	94	118	120	120	99–121
**Ventral**	167	141	140	141	162	154	140	141	132	95–124
**Ventral/dorsal length**	2.6	–	1.6	1.6	1.6	1.6	1.2	1.2	1.1	1.0

Transverse, oval male pores are in line of ventral chaetae of segment X and XI, about midway between chaetae and posterior septum (Fig. 1A). Female pores open just below the lateral line, in intersegment 12/13. Inconspicuous, round spermathecal pores open behind, and in line with the ventral chaetae in XII–XIII.

Pharynx developed mainly dorsally and laterally, in segments II and III. Pharyngeal glands well developed dorsally and ventrally in IV–VI, usually extending ventrally into VII. Chloragogenous tissue well developed from VII backwards. A supra-intestinal vessel may appear differentiated from the perivisceral sinus (Fig. 2E) beginning in XIV; this is not evident after the dorsal vessel joins the gut in about XX. One pair of simple commissural blood vessels join dorsal and ventral vessels in anterior segments to about XV; those in XII may loop into the egg sacs (Fig. 2D). Lateral blood vessels absent from posterior segments except for 1–2 very short lobes on dorsal vessel in about the posterior 1/4 of the body (Fig. 1E). Nephridia usually paired in VII and VIII, and paired, single, or absent in segments posterior to XIII; efferent ducts simple, mostly limited to ventral half of body, without vesicles at nephridiopores. Sperm sacs extend anteriorly to VIII or IX, and backwards as far as XXII. Egg sacs may extend to 2 or 3 segments beyond sperm sac; when eggs have partially completed vitellogenesis, egg sacs shorter, not extending beyond sperm sacs, to XIII or XIV.

Two pairs small testes, in segments X and XI; one pair elongate ovaries in XII, extending through XIII. Female funnels large, attached to the septum and opening in intersegment 12/13. Two pairs spermathecae, the first in the post-atrial segment (typically XII), and the second in the post-ovarian segment (typically XIII) (Figs 1C, 2F).

A single vas deferens per atrium (prosoporous condition), sperm funnels located on the septa of intersegments 10/11 and 11/12 (posterior septa of atrial segments), but folded back into the next segment. Vasa deferentia long (about 700 μm), penetrating the posterior septa, and forming a long, convoluted loop within each post-atrial segment (Fig. 1C). Vasa deferentia narrow (12–16 μm diameter) and transparent, each joining the atrium at the ectal (or basal) part of the ampulla (adjacent to the atrial duct), and running under the atrial musculature to about the middle part of the ampulla, where it opens into the atrial lumen (Fig. 1C, D). Atria petiolate, extending medially from male pore, with nearly spherical ampulla (140–210 μm diameter, slightly longer than wide) and tubular ectal duct (Fig. 1D). Ampullar musculature very thick (40–50 μm), organized in many intercrossing layers (Fig. 2H–J). Atrial ampulla with very thin epithelium, and covered by a thin (up to 5 μm) layer of cells and prostate glands formed by elongate-petiolate clusters of cells; each gland is pedunculate with a narrow extension penetrating the atrial musculature (Fig. 2I). Atrial duct tubular (17–24 μm diameter, 90–110 μm long), composed of an epithelium surrounded by loose, indistinct musculature, extending into a type-1 penis (Fig. 17, in Rodriguez and Giani 1994) within a deep penial sac (120–230 μm deep) (Figs 1C, D, 2M), and associated with retractor muscles extending dorso-laterally to the body wall. Penis length 90–110 μm; the broad, ental part forms a distinct epithelial tube which disappears ectally; the middle portion is surrounded by a ring of what appears to be circular musculature; and the ectal part is sharply acuminate, covered by a thin (ca.1 μm), cuticular layer.

The spermathecae have a narrow duct and an irregular, sacciform ampulla. Spermathecal duct fusiform, (30–45 μm maximum diameter), formed by columnar epithelium, a thin (about 2 μm) muscle layer, and with a wide lumen except at the pore; ental end of the duct prolonged into a narrow neck (12–20 μm diameter) which joins the ampulla (Figs 1C, 2L). Duct sharply narrowed at the pore, with a short sphincter surrounded by a circular muscle layer (Figs 1C, 2K). Ampulla in two parts, a short ectal section (60–90 μm long by 35–46 μm wide), lined with irregular epithelium, and a much larger ental part (320–480 μm by 130–250 µm), which is lined by columnar, vacuolated epithelium, up to 35 μm thick (Fig. 2G, K). Sperm within the spermathecae loose and unordered; epithelial vacuoles not obviously containing resorbed sperm. All spermathecae similar in size; ampullae of mated worms may extend into adjacent segments.

#### Anomalies.

Two specimens had the entire sequence of reproductive organs in segments VII–X, with the clitellum in VII–XI instead of the usual X–XIV; apparently an anterior shift of three segments. These aberrant worms appeared normal in other respects, except that nephridia were not present in VII and VIII.

#### Taxonomic remarks.

**Figure 3.**
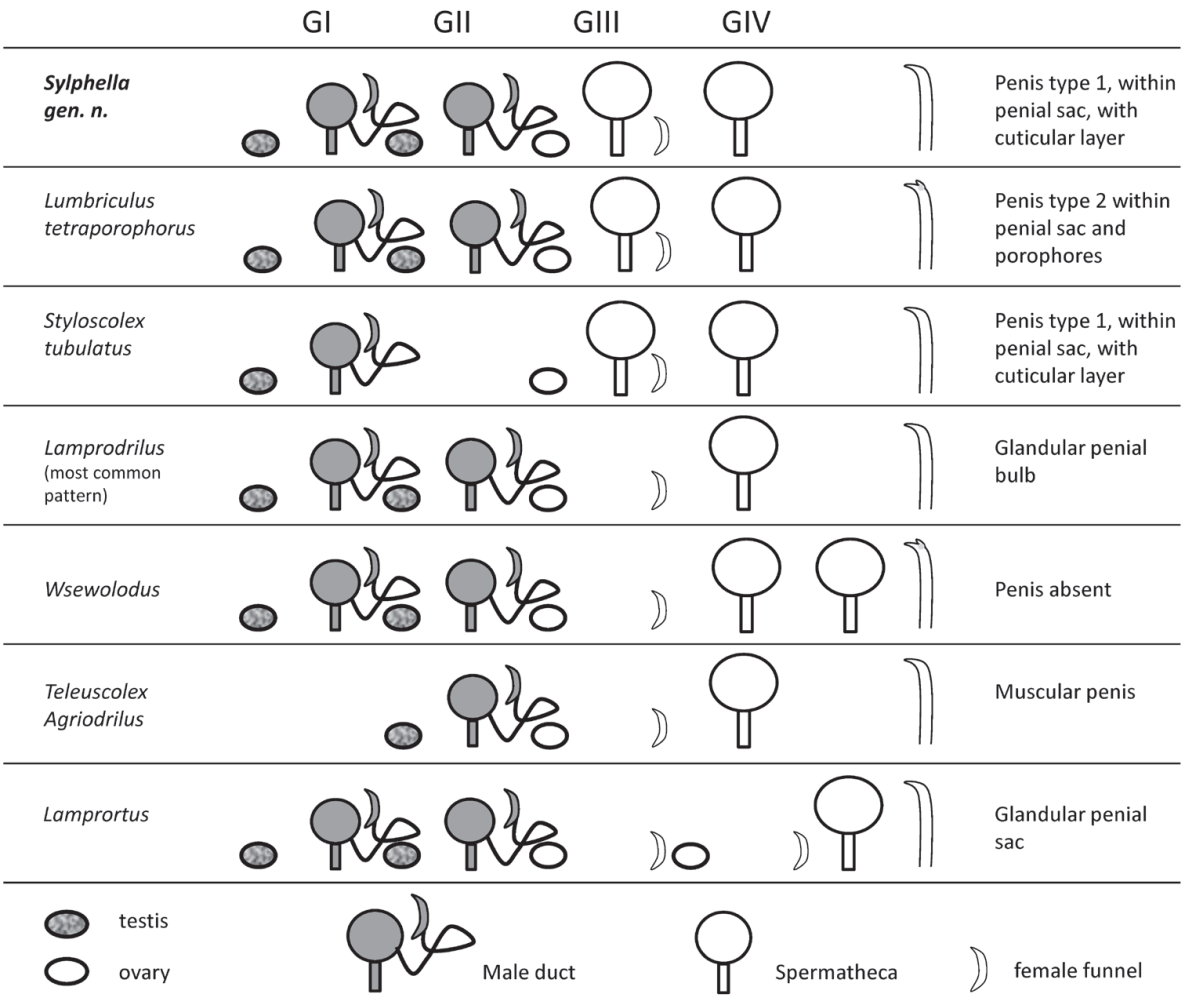
Comparative schema of the reproductive system and chaetae in the new genus *Sylphella* and other related prosoporous lumbriculid genera. Type-1 and type-2 penes as described by Rodriguez and Giani (1987) (see text).

The combination of multiple atrial segments, prosopore male ducts in the testicular segments (GI and GII, see Brinkhurst 1991), and postatrial spermathecae in *Sylphella puccoon* gen. n., sp. n. is shared with the monotypic genera *Lamprortus* Rodriguez, 1994 (in Brinkhurst et al. 1994) and *Wsewolodus* Semernoy, 2004 (Fig. 3). Additionally, this arrangement of reproductive organs occurs in some species (or variants) of *Lumbriculus* Grube, 1844 and *Lamprodrilus* Michaelsen, 1901 (*Teleuscolex* and *Agriodrilus* included). *Lamprortus* is well distinguished from other lumbriculids by its hologyny, i.e., by the possession of 2 pairs of testes and 2 pairs of ovaries. *Lamprortus* and most *Lamprodrilus* species have only one spermathecal segment, although variants of *Lamprodrilus mrazeki* Hrabě, 1928 and *Lamprodrilus satyriscus* Michaelsen, 1901 may have two or more pairs of spermathecae. Almost all lumbriculids with two atrial segments differ from *Sylphella* gen. n. in having one intervening segment between the last atrial segment and the first spermathecal segment (Fig. 3). Thus, relative to the gonads, the first spermathecal segment is in the first post-ovarian segment (GIV in *Lamprodrilus* and *Wsewolodus*, and behind GIV in *Lamprortus*). In *Sylphella*, the first spermathecae are in the ovarian segment (GIII). The genus *Lumbriculus* is highly variable not only in number but also in the position of the spermathecae, but usually more than two pairs open laterally, either at the dorsal or ventral side of the body. The closest match to *Sylphella* is *Lumbriculus tetraporophorus* Popchenko, 1976a, but that species is distinguished from *Sylphella* by typical *Lumbriculus* characters, including bifid chaetae, a plexus of anterior commissural blood vessels, and *Lumbriculus*-type male reproductive organs, with a pyriform atrium and penial sac ending in a porophore (see Table 2).

**Table 2.** Taxonomic characters of the reproductive system that distinguish *Lumbriculus tetraporophorus* Popchenko, 1976 from *Sylphella puccoon* gen. n., sp. n.

Characters	*Lumbriculus tetraporophorus*	*Sylphella puccoon*
**Male porophores**	2 pairs, prominent, conical (210 μm high), formed by concentric muscle ridges	Absent
**Atrial ampulla**	Pear-shaped (250×510 μm)	Nearly spherical (140–210 μm Ø)
**Atrial duct**	170 μm long, wider in the middle	Cylindrical (90–110 μm long). Penial sac 120–130 μm
**Atrial musculature**	48–51 μm, in 2 orthogonal layers, circular muscle 34 μm thick	40–50 μm intercrossing fibers in many indistinct layers
**Penis**	140–170 μm long, with tapered end (probably typical *Lumbriculus* type of extrudable lining cells in the atrial duct)	90–110 μm long, tapered end of atrial duct sharply acuminate, ectally covered by cuticle
**Prostatic cells**	Diffuse (*rykhlym*)	In petiolate clusters of cells
**Vas deferens**	Prosoporous, 18–20 μm Ø, joining entally, barely penetrating next segment	Prosoporous, (12–16 μm Ø, joining ectally, forming a loop in next segment
**Female pores**	ventral	lateral

The tetrathecate condition, with paired spermathecae in the first two postatrial segments, is a feature shared with some species in the semiprosoporous lumbriculid genera *Trichodrilus* Claparède, 1862 and *Eremidrilus* Fend & Rodriguez, 2003. However, the presence of two pairs of prosoporous atria in *Sylphella* suggests that a close phylogenetic relationship with these genera is improbable.

The general form of the atria bears a slight resemblance to some Palearctic species of the genus *Trichodrilus* having petiolate atria, spherical and very muscular atrial ampullae, and two pairs of spermathecae (e.g., *Trichodrilus aporophorus* Popchenko, 1976b, *Trichodrilus claparedei* Hrabě, 1937). *Bichaeta sanguinea* Bretscher, 1990 also has a spherical and very muscular atrium, but lacks an atrial duct. In contrast, genera resembling *Sylphella* in the arrangement of reproductive organs (*Lamprodrilus*, *Lamprortus*, *Wsewolodus* and *Lumbriculus*) tend to have elongate atria.

The atrial musculature in *Sylphella* consists of many small, cross-hatched layers, similar to some other lumbriculids, such as *Trichodrilus longipenis* Giani & Rodriguez, 1994. Details of atrial musculature are usually not given in lumbriculid diagnoses, but where described, the atrial muscle fibers show a simpler organization (parallel or two opposing layers) in the related genera. The *Sylphella* arrangement of atrial muscles should be distinguished from the simple crossed musculature in *Lumbriculus* species, which consists of only two perpendicular layers; however, it bears some resemblance to the many diagonally arranged layers in some *Eclipidrilus* Eisen species (Fend 2005).

The penis in *Sylphella puccoon* is similar to that described for *Styloscolex japonicus* Yamaguchi, 1937 in its basic structure, as well as in the presence of a smooth, rigid cuticular layer (sheath) on the ectal end (Fig. 2M, N). *Styloscolex* Michaelsen, 1901 has an intervening segment between the testicular and the ovarian segments, an autapormorphy that separates this genus from other lumbriculids. Several other *Styloscolex* characters, including pre-atrial spermathecae in most species, elongate atria in a single segment, and a forward shift in reproductive organs (Brinkhurst 1989) suggest that *Styloscolex* is probably not closely related to *Sylphella*.

*Lamprortus* and most *Lamprodrilus* species also have a type-1 penis (i.e., an extension of the atrial duct within a fold of the ventral body wall, see Rodriguez and Giani 1994), but these usually have a characteristic structure, being associated with a large mass of glands. Some *Lamprodrilus* species also have muscular penial bulbs. *Lumbriculus* species have a type-2 penis (i.e., formed in part by elongation of atrial lining cells) within a penial sac formed by very thick, columnar epithelium (see Hesse 1902 for *Lumbriculus variegatus*). Penes are absent in *Wsewolodus*.

Enlarged ventral chaetae in anterior segments occur to some degree in many lumbriculids, but the difference is well marked in several *Trichodrilus* species (see Rodriguez and Giani 1994), *Lamprodrilus inflatus* Michaelsen, 1905, and *Stylodrilus mirus* (Chekanovskaya, 1956).

#### Ecological remarks.

*Sylphella puccoon* gen. n., sp. n. has only been collected during winter months from a single, small seep that is a tributary of Pokeberry Creek, North Carolina. A large number of similar seeps were investigated by one of the authors (D. Lenat) adjacent to Pokeberry Creek, but *Sylphella* was limited to a 10-m reach of the largest seep (1 meter wide). The small streams in this area go completely dry during summer months, due a combination of clay soils and seasonal rainfall patterns. The dominant macroinvertebrates in these seeps were the isopod *Caecidotea forbesi* (William), the amphipod *Crangonyx* sp. Bate, and chironomids. The mayfly genera *Callibaetis* Eaton and *Leptophlebia* Westwood can be abundant, but other EPT taxa were sparse. Other oligochaetes at this site include *Rhynchelmis bolinensis* Fend & Lenat (the type locality), Eclipidrilus cf. fontanus, *Rhyacodrilus propiporus* Rodriguez & Fend, and an undescribed lumbriculid of unknown generic attribution.

### Cookidrilus pocosinus sp. n.

Taxon classificationAnimaliaLumbriculidaLumbriculidae

http://zoobank.org/D72DE213-2FCC-4B0C-A696-FEED022939D0

Figs 4 and 5

#### Holotype.

USNM 1251699: a dissected specimen, stained in Harris’ hematoxylin and mounted in Canada balsam (collected 4 March 2011).

#### Paratypes.

USNM 1251700-1251702: from the type locality, 22 Feb 2011, 1 dissected; 4 Mar 2011, 1 whole-mounted; Pettiford Creek, at Millis Road, Carteret County, North Carolina, USA, 15 Mar 2007, 1 whole mount. MNCN 16.03/3084: from the type locality, 22 February 2011, 1 dissected, stained in Harris’ hematoxylin and mounted in Canada balsam, and 1 histologically sectioned, stained with hematoxylin-eosin. CASIZ 197899: Pettiford Creek, 15 Mar 2007, 1 whole mount.

#### Type locality.

Lake Run, outlet stream draining Little Singletary Lake at SR 1325, in Bladen County, North Carolina, USA.

#### Etymology.

The specific name refers to *pocosin*, “swamp-on-a-hill” in the Algonquin Indian language. Most specimens were collected in two sites draining pocosin areas.

#### Other material.

From the type locality, 22 Feb 2011, 4 whole mounts, 1 dissected, 1 sagittally sectioned. Pettiford Creek, at Millis Road, Carteret County, North Carolina, USA, 22 Apr 2008, 1 whole mount; 5 Apr 2010, 2 dissected. Drowning Creek at State Road 1004, Moore County, North Carolina, 12 Jan 2009, 1 whole mount. Anderson Creek at SR 2031, Harnett County, North Carolina, 27 Jun 2011, 1 whole mount. All specimens (including the type series) collected by D.R. Lenat.

#### Description.

**Figure 4.**
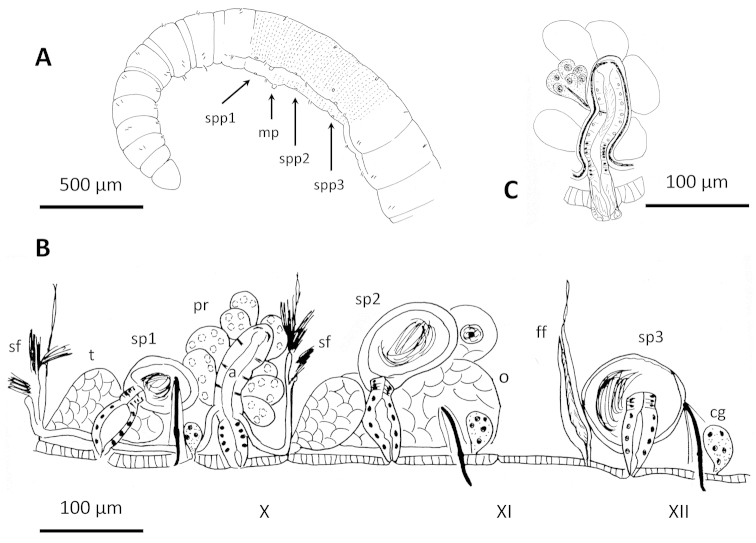
Drawings of *Cookidrilus pocosinus* sp. n. **A** anterior body region **B** reproductive organs **C** detail of the atrium showing the vasa deferentia junction and prostatic cell clusters.

**Figure 5.**
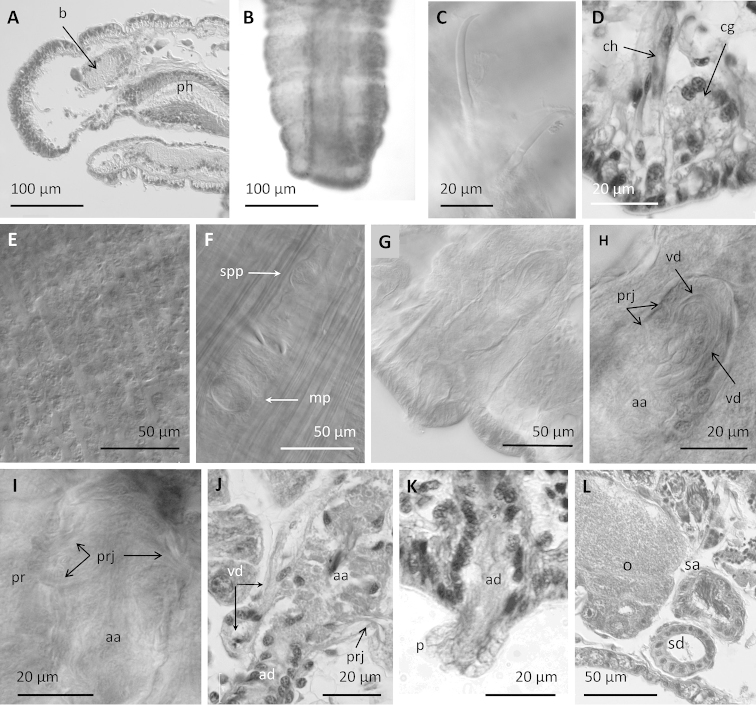
*Cookidrilus pocosinus* sp. n. **A** Anterior region of the body **B** caudal region with pygidium **C** chaetae **D** chaetal gland behind ventral bundle of chaetae **E** clitellum **F** spermathecal and male pores in front of and behind ventral chaetae of segment X **G** atrium **H** atrial ampulla showing apical junction of vasa deferentia **I** atrial ampulla showing junction of prostatic cell clusters **J** basal junction of vas deferens to atrial ampulla **K** detail of atrial duct and protruded penis **L** third spermatheca behind the female segment. **A, D, J, K, L** histological sections, other photographs from stained whole mounts or dissected specimens.

Number of segments 53–71. Diameter of the body 279–342 µm in segment VIII and 360–441 µm at the clitellum. Prostomium round, 120–154 µm long. Brain back to intersegment 2/3. Secondary annulation (narrow ring in anterior part of segment) well marked from segment VI to IX, not always visible in the postclitellar region, but evident in the caudal region of the body (Figs 4A, 5B). Epidermis in anterior segments 10–16 µm high. Clitellum from segment X to XII, with epithelium 16–34 µm high, formed by small glandular cells arranged in regular transverse rows (Fig. 5E). Chaetae sigmoid, simple-pointed (Fig. 5C), length about equal in dorsal and ventral bundles, shortest in segment II (56–62 µm), progressively longer to the middle of the body (68–82 µm long), and gradually shorter to the end of the body (down to 66 µm). A chaetal gland behind chaetal bundles, conspicuous in anterior segments, smaller posteriorly (Figs 4B, 5D). Nodulus at about 0.3–0.4 from the distal end. Pygidium normally formed (Fig. 5B). Male pores located behind and in line of ventral chaetae of segment X (Fig. 5F). Female pores open in the line of ventral chaetae, in intersegment 11/12. Spermathecal pores opening midway between ventral chaetae and anterior septum, in line with the ventral chaetae, in atrial and 2 postatrial segments. In most sexually mature, fixed individuals, the ventral region of clitellar region is concave with prominent lateral margins (saddle shaped clitellum).

Pharynx developed mainly dorsally, in segments II and III. Pharyngeal glands in last part of IV, and well developed in V and VI, dorsally and laterally. Chloragogenous tissue starting in the hind part of VI and well developed from VII backwards. Nephridia present on at least one side in VII in some specimens; most specimens have at least one nephridium in XIII, and in a few posterior segments. Nephridiopores inconspicuous, without vesicles; nephridial duct very thin and transparent. Sperm sac extends forward to VIII, and backward to XII–XV. Egg sac back to XIII–XVII.

Two pairs testes, in segments IX and X, and one pair ovaries in segment XI. Two vasa deferentia per atrium (semiprosoporous condition), originating in sperm funnels located in the septa of intersegment 9/10 and 10/11, respectively. Posterior vas deferens not entering segment XI. Vasa deferentia very narrow (8–14 µm diameter), joining the atrium in the ectal (or basal) part of the ampulla, and running through the atrial musculature to the apical part of the atrium, where they open to the atrial lumen (Figs 4B, C, 5H, J). Atrium tubular, with elongated ampulla (86–120 µm long, 26–36 µm diameter) and short duct (34–40 µm long, 24–26 µm diameter). Atrial muscle layer thin, 2–3 μm thick. The columnar inner epithelium of the atrial duct can extend beyond the male pore forming a short, protrusible penis (less than 40 µm long, when protruded) formed by the extension of lining cells of the atrial duct (Figs 4C, 5G, K), extended cells may appear vacuolated. Atrial ampulla with ciliated lumen, and covered by 8–10 well-separated prostate glands formed by clusters of cells, each of which tapers to form a narrow stalk before joining the ampulla (Figs 4C, 5I, J).

Female funnels large, attached to the septum and opening in intersegment 11/12 (Fig. 4B). Three pairs of spermathecae, the first in the atrial segment (X), and the next in the ovarian (XI) and post ovarian (XII) segments. The spermathecae are formed by a bottle-shaped duct (34–86 µm long, 22–38 µm maximum diameter) and an oval ampulla (54–120 µm maximum diameter and 34–90 µm minimum diameter), filled by loose sperm (Figs 4B, 5L). In several of the examined specimens, the ampullar epithelium appeared very much vacuolized, with some vacuoles containing resorbed sperm.

#### Taxonomic remarks.

*Cookidrilus pocosinus* sp. n. has been ascribed to the genus *Cookidrilus* Rodriguez & Giani, 1987 based on the main diagnostic characters of the genus: 2 pair testes and one pair ovaries, two (anterior and posterior) vasa deferentia joining each atrium, one pair spermathecae in the atrial segment, and subsequent pairs of spermathecae in postatrial segments (Rodriguez and Giani 1987). The groundwater lumbriculid genus *Cookidrilus* was originally described from the Labouiche Cave in southern France. Since then, another species has been described from the hyporheos of Lachein Creek, a karstic stream in the same geographic region (Route et al. 2004). In the present study, a third species is described from coastal plain habitats of North Carolina (USA), mostly from acidic swamp streams.

In Table 3, we have summarised the main morphological features that distinguish the three species of the genus *Cookidrilus*. The new species is closer to the type species of the genus, *Cookidrilus speluncaeus* Rodriguez & Giani, 1987, based on the presence of three pairs of spermathecae instead of only two pairs in *Cookidrilus ruffoi* Giani et al., 2004 (in Route et al. 2004). However, it resembles *Cookidrilus ruffoi* in the structure of the prostatic cell layer, which is organised in well-separated clusters that join the atrial ampulla by distinct stalks. The new Nearctic species *Cookidrilus pocosinus* is distinguished from both European species by the singular position of the spermathecal pores in front of the ventral chaetae, instead of behind the chaetae (the most common position in lumbriculids). The genus *Cookidrilus*, previously amended by Route et al. (2004), is now further amended to include some additional diagnostic features.

**Table 3.** Morphological characters of the three known species of the genus *Cookidrilus* Rodriguez & Giani, 1987.

*Cookidrilus*	*speluncaeus*	*ruffoi*	*pocosinus* sp. n.
**Body diameter**	350 µm at clitellum	583–633 µm	360–441 µm at clitellum
**Double annulation begins**	In III	–	In VI
**Prostomium form and length**	Round, 76–83 µm	Often wrinkled, 305–400 µm	Round, 120–154 µm
**Clitellum**	X–XII	Poorly developed, in X–XII	X–XII
**Pharynx**	Dorsal and ventrally well developed, in II–IV	Dorsal and ventrally well developed, back to VII, VIII	Typical dorsal pad, in II–III
**Pharyngeal glands**	IV–VIII	III–VII (VIII)	IV (posterior)–VI
**Chaetae, length**	73–82 µm in ante-clitellar region	105–112 µm in II, 174–236 µm in anterior to middle region	56–62 µm in II, 68–82 µm in anterior to middle region
**Posterior body region**	Not modified	Evaginable tube	Not modified
**Spermathecae, number and position of pores**	3 pairs, pores behind ventral chaetae	2 pairs, pores behind ventral chaetae	3 pairs, pores in front of ventral chaetae
**Spermathecal ducts, form and length**	Tubular, short ducts 44–76 µm	Bottle shaped, 115–143 µm	Bottle shaped, short ducts, 34–86 µm
**Spermathecal ampulla**	The first is small, the third penetrates XIII	Do not penetrate other segments	The first smaller. Do not penetrate other segments
**Atrium**	In X	In X	In X
**Atrial ampulla, shape and size**	Pyriform, 71 µm long, 48 µm Ø	Pyriform, 207 µm long, 161 µm Ø	Tubular, 86–110 µm long, 26–30 µm Ø
**Atrial duct length**	29 µm	84 µm	34–40 µm
**Penis**	Simple pore	Protrusible penis	Protrusible penis
**Prostate layer**	Dense, diffuse layer	3–4 clusters of cells	8–10 clusters of cells
**Atrial muscular layer**	4 µm thick	16–20 µm thick	2–3 µm thick
**Vas deferens diameter / junction to the atrium**	11 µm / apical	15–20 µm / lateral	8–14 µm / at the base of the ampulla (opening apically)
**Posterior vas deferens**	Penetrates 10/11	Penetrates 10/11	Does not penetrate 10/11

In the original description of the genus, Rodriguez and Giani (1987) discussed the taxonomic relationships of *Cookidrilus* with other lumbriculids (*Kincaidiana* Altman, 1936 and *Guestphalinus* Michaelsen, 1933) having a pair of spermathecae in the atrial segment. *Kincaidiana hexatheca* Altman, 1936 is endemic to North America where there are also representatives of the genus *Guestphalinus* (S. Fend, unpublished data). However, although the former has a similar arrangement of spermathecae to *Cookidrilus speluncaeus*, a combination of characters clearly distinguishes it from *Cookidrilus*: a proboscis, a forward shift of reproductive organs, a single pair of testes, and one prosoporous vas deferens per atrium (Fend 2009). In addition, morphology of the atria, spermathecae, and chaetae does not resemble that of the known *Cookidrilus* species. *Guestphalinus* is semiprosoporous, but has only one pair of spermathecae, and like *Kincaidiana*, has a proboscis. *Guestphalinus* also has a forward shift in reproductive organs relative to the position in *Cookidrilus*.

The presence of penis may be a common generic character in *Cookidrilus*, since it is only absent in the type species, *Cookidrilus speluncaeus*. On the other hand, *Cookidrilus ruffoi* differs in the number of spermathecae. The analysis of lumbriculid genera performed by Brinkhurst (1989) stated that characters related to number and placement of the spermathecae (characters 7 and 9 in that analysis) were subject to changes/reversals in the resulting phylogenetic tree, and such variations are probably not highly significant. Thus, variation in number of spermathecal segments within *Cookidrilus* (3 in two species, versus 2 in *Cookidrilus ruffoi*) is not extraordinary, as similar variation occurs in other lumbriculid genera such as *Trichodrilus* and *Rhynchelmis* Hoffmeister, 1843 (see Cook 1971).

The position of spermathecal pores in front of the ventral chaetae is an unusual feature of the new *Cookidrilus* species. Spermathecal pores in lumbriculids are usually placed behind the chaetae of the corresponding segment, and in the other 2 species of *Cookidrilus*, even the first spermatheca opens in the narrow space between the ventral chaetae and the male pores. This character is shared with several Nearctic lumbriculids: *Kincaidiana hexatheca* (for the first pair of spermathecae), some *Rhynchelmis* species (in Fend and Brinkhurst 2000), and *Eclipidrilus pacificus* Fend, 2005.

#### Ecological remarks.

*Cookidrilus pocosinus* sp. n. appears to have a life cycle adapted to seasonal drying of surface flow. This is the first record of *Cookidrilus* in North America, and it is also the first report of the genus in a non-subterranean habitat. This species has been found so far in four North Carolina streams, but almost all specimens were from Lake Run and Pettiford Creek. These streams are both located in the southern Coastal Plain, in relatively undisturbed watersheds, and drain pocosin areas with peat soils. Both streams have extremely low pH values (often less than 4.0), very low conductivity, and dry up completely during summer droughts. In Lake Run, most specimens were found in shaded sections, in midstream areas with both good flow and a fine sand substrate. In Pettiford Creek, the substrate consisted of fine sand covered by a layer of organic debris. These data indicate that *Cookidrilus pocosinus* is usually associated with very low pH, although single specimens were collected from a seasonally inundated side channel of Drowning Creek, and from the main channel of Anderson Creek. Both of those streams have average pH values near 5.5.

#### Diagnosis of the genus

(amended by Route et al. 2004, and modified here with additions in italics): **Type species.** *Cookidrilus speluncaeus* Rodriguez & Giani, 1987.

Chaetae sigmoid, simple-pointed. One pair male pores in segment X (*the second testis-bearing segment*), behind and in line with the ventral chaetae. *Two or three pairs spermathecae; first pair in the atrial segment, anterior to male pores, and* one pair *in the first, or in the first and second postatrial segments*. Two pair testes, in IX and X. *One pair atria in the second testicular segment. Semiprosoporous male duct, two vasa deferentia per atrium. Prostatic cells either in a simple diffuse layer or forming discrete clusters*. One pair ovaries located in the first postatrial segment (XI).

### Stylodrilus coreyi sp. n.

Taxon classificationAnimaliaLumbriculidaLumbriculidae

http://zoobank.org/361604D8-0420-44CE-8F91-BFBB133A1510

Figs 6 and 7

#### Holotype.

USNM 1251703: A whole-mounted specimen in Canada balsam (collected 19 Jan 2010).

#### Paratypes.

USNM 1251704-1251707: 17 Feb 2007, 1 whole mount; 19 Jan 2010, 2 whole mounts; 5 Apr 2010, 1 dissected. MNCN 16.03/3085: 19 Jan 2010, 1 dissected and 1 histologically sectioned; 5 Apr 2010, 1 whole mount, stained in borax carmine. CASIZ 197900: 16 Feb 2011, 2 dissected. All from the type locality.

#### Type locality.

Pettiford Creek at Millis Road, Carteret County, North Carolina, USA.

#### Etymology.

This species is named in honor of Jesse Edward (Ed) Corey III, an Inventory Biologist at the North Carolina Division of Parks and Recreation. We celebrate Ed’s unwavering interest in all animals and plants, including our beloved oligochaete worms.

#### Other material.

From the type locality: 17 Feb 2007, 1 dissected. 30 Sep 2009, 7 whole mounts, 1 dissected. 19 Jan 2010, 5 whole mounts, 2 dissected, 3 sectioned (2 sagittal, 1 transverse), 2 in alcohol. 5 Apr 2010, 6 whole mounts, 2 dissected. 16 Feb 2011, 1 whole mount, 10 in alcohol. Floodplain seeps in Drowning Creek floodplain at State Road 1004, Moore County, North Carolina: 31 Dec 2008, 2 whole mounts. 12 Jan 2009, 2 whole mounts, 5 dissected. 17 Feb 2011, 1 whole mount. All specimens (including the type series) collected by D.R. Lenat.

#### Description.

**Figure 6.**
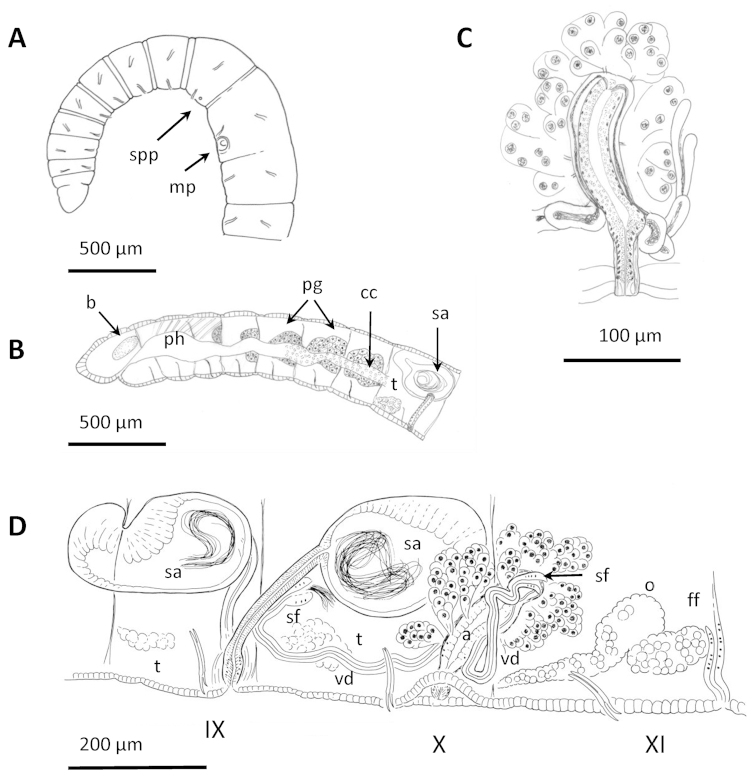
Drawings of *Stylodrilus coreyi* sp. n. **A–B** Anterior part of the body showing double annulation and genital pores (**A**) and digestive tract with associated glands (**B**) **C** details of male duct **D** reproductive organs.

**Figure 7.**
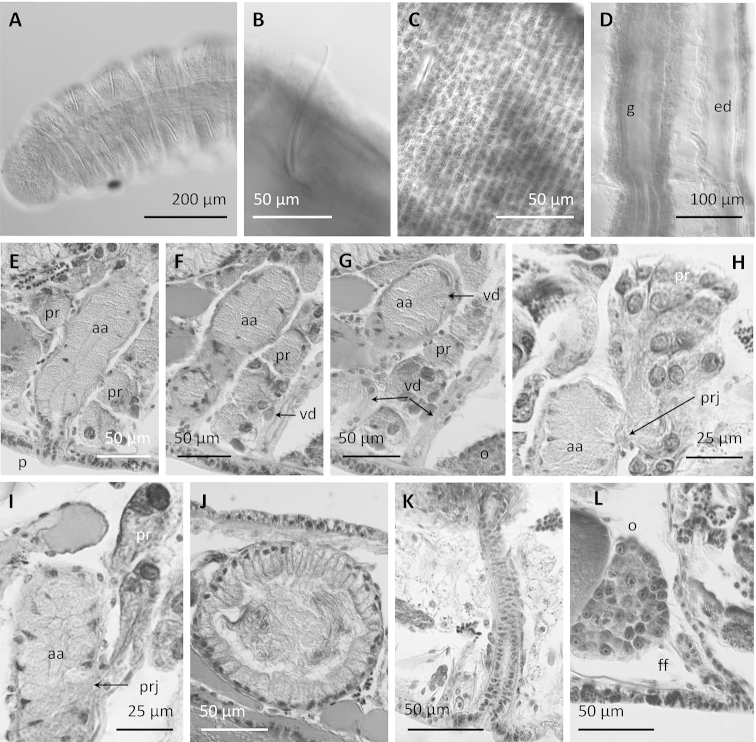
*Stylodrilus coreyi* sp. n. **A** Anterior part of the body, B: simple-pointed chaeta **C** clitellum **D** nephridial efferent duct in ventral part of posterior segment (anterior part facing up) **E–G** consecutive histological sections of male duct **H–I** details of prostatic glands and connection to atrial ampulla **J** spermathecal ampulla **K** spermathecal duct **L** female funnel. **E–L** histological sections, other photographs from stained whole mounts or dissected specimens.

Number of segments 53–69. In 27 unmounted specimens, body length 11.7–14.2 mm, diameter of the body in segment VIII, 240–585 µm (mean 379 µm); maximum diameter in the clitellar region to 760 µm (mean 467 µm); midbody diameter 330–630 µm (mean 429 µm). Prostomium round or conical, 142–196 µm long (Figs 6A, 7A). Brain deeply lobed, back to septum 2/3. Clitellum saddle-shaped, formed by cells in distinct rows (Fig. 7C), extending from the anterior part of segment X (from the level of chaetae) to the end of segment XII. Epidermis 6–17 µm high in anterior segments, and up to 23–34 µm in the clitellum; 25–32 µm high in the prostomium. Secondary annulation (a narrow ring in anterior part of segment) usually in IV to IX.

Chaetae simple-pointed (Fig. 7B), nodulus at 0.3 (rarely at 0.4) from the ectal end, of similar size in dorsal and ventral bundles or slightly longer in ventral bundles; smaller in segment II (63–70 µm), length increasing in the anterior segments to segment VIII (73–116 µm), and usually smaller in the posterior part of the body (71–111 µm, down to 63 µm).

One pair spermathecal pores in segment IX and one pair male pores in segment X, in line with and behind the ventral bundles of chaetae (one specimen from Drowning Creek regenerating the anterior part of the body, with spermathecal pores in VII and male pores in VIII). One pair female pores in the intersegment 11/12.

Pharyngeal pad well-developed dorsally, usually extending through IV; pharyngeal glands from the posterior part of segment IV back to VIII, dorso-lateral and ventral to the gut in segments V to VIII (Fig. 6B). Chloragogen cells covering the gut from the posterior part of segment VI onwards. Nephridia with long efferent ducts observed in segment VII and in some postclitellar segments, tubular shaped, running ventrally through several segments (Fig. 7D); nephridiopores without vesicles, in front of ventral chaetae. Lateral blood vessels absent in posterior segments. Two pairs testes, in anterior part of segments IX and X, and one pair ovaries in segment XI. Sperm sacs back to segment XV or XVI (never observed extending forward), and egg sacs back to XVI or XVII.

Semiprosoporous male ducts, with one anterior vas deferens attached to the sperm funnel in intersegment 9/10, and the posterior one to the funnel in 10/11, the anterior being longer (280–480 µm) than the posterior (215–300 µm). Both funnels appear deflected backward, somewhat behind their respective septa when full of sperm. Vasa deferentia (15)20–28 µm in diameter, to 34 µm close to the sperm funnel. Posterior vas deferens does not enter postatrial segment (Figs 6D, 7G). Atrium elongate (176–390 µm total length, including the penis), with the ampulla (120–184 µm long, 43–70 µm maximum diameter) usually restricted to segment X, but in some individuals passing into segment XI. Several discrete clusters of prostatic cells (44–100 µm high) join the atrium by distinct stalks that traverse the atrial musculature (Fig. 7E–I). Atrium length 0.45–0.70 (usually c. 0.50) times the body diameter at the clitellum. Short atrial duct not distinctly separated from the ampulla, narrowing to about 24 µm wide, the male pore on a short penis (25 µm long), in a shallow fold of the body wall. Several dorso-ventral muscular strands are associated with the male pore. Atrial epithelium very granulated, 14–19 µm high, and atrial lumen ciliated; atrial musculature thin (4–6 µm thick). Vasa deferentia join the atrium at about the basal one third, and run under the atrial musculature to the most apical part of the ampulla, where they open to the atrial lumen (Figs 6C, 7G).

One pair spermathecae, with ampullae typically located in segments IX and X, oval to nearly spherical (174–331 µm diameter, 205–348 µm long), containing a mass of loose sperm in the ectal part, sometimes together with amorphous material (Figs 6D, 7J). Ampulla with thin epithelium in ectal part (about 5–10 μm); epithelium with large, irregular cells (to over 50 μm), which may fill the lumen in ental part; no sorptive vacuoles were observed. Spermathecal duct long (150–247 µm) and relatively thin (22–31 µm diameter), slightly widening at ectal end up to 42 µm; with narrow, columnar cells and thin (<5 μm) muscle coat. One pair female funnels open ventrally in intersegment 11/12 (Figs 6D, 7L).

Worms from Downing Cr are generally larger than those from Pettiford Cr (see Table 4), but morphology is otherwise similar.

**Table 4.** Comparison of morphological features in two study populations of *Stylodrilus coreyi* sp. n.

Population	No. segments	Body Ø in VIII µm	Chaetae length µm	Atrium length µm	Atrium Ø µm	Spth duct length µm	Spth ampulla length µm	Spth ampulla Ø µm
**Pettiford Creek**	53–65	240–420	63–105	176–248	40–63	c.163–225	205–206	174–179
**Drowning Creek**	55–69	390–585	81–120	266–390	54–74	150–247	239–348	198–331

#### Taxonomic remarks.

*Stylodrilus coreyi* sp. n. conforms to the general diagnosis of the genus *Stylodrilus* Claparède, 1862 (see Rodriguez and Coates 1996), which includes most known lumbriculid species with 2 pairs of testes and one pair of ovaries, one pair of spermathecae in the first testicular segment, and one pair of semiprosoporous male ducts in the second testicular segment. According to Hrabě (1929, 1970), the genus *Bythonomus* Grube, 1880, which had the same arrangement of reproductive organs, was restricted to those species with all chaetae simple-pointed, 2 pairs of branched lateral blood vessels (sometimes only bifurcate or absent), tubular or oval atria, and vasa deferentia opening basally (ectally) to the atrium. *Bythonomus* was classified as junior synonym of *Stylodrilus* by Brinkhurst (1965), a decision that was refuted by Hrabě (1970), and still divides taxonomists in the present. For example, Giani and Martínez-Ansemil (1984) accepted the synonymy, and used the characters of *Stylodrilus glandulosus* as an example to invalidate *Bythonomus* as a genus; this view is actually supported by most authors (e.g., Rodriguez 1988, Timm 2009). However, Kaygorodova and Martin (2008), still supported a distinction between *Bythonomus* and *Stylodrilus*, based on the shape of chaetae. Until the taxonomic status of *Stylodrilus* is clarified by molecular analyses, we accept the synonymy, since several species of the *Stylodrilus* complex have a mixture of *Bythonomus*-like and *Stylodrilus*-like characters. In the present case, the new species has simple-pointed chaetae and tubular atria, but no posterior lateral blood vessels have been observed, and although the junction of vasa deferentia is basal, the opening to the atrial lumen is completely apical. The junction of the vasa deferentia to the atrium is not always easily defined as basal or apical in the “*Bythonomus* group”, but rather it occurs in every possible position from basal to apical (Brinkhurst and Wetzel 1984). Besides, the atrial shape can be difficult to categorize in species with long atrial ducts that gradually widen toward the ampulla.

*Stylodrilus coreyi* sp. n. belongs to a group of *Stylodrilus* species with simple-pointed chaetae, elongate atrium, and posterior vas deferens not forming a loop in the postatrial segment (Table 5). Within this group, *Stylodrilus coreyi* is distinguished by several features of the male duct: atrium length about half (0.5–0.7) times the diameter of the body at the clitellum; a very short, barely differentiated atrial duct forming a short penis within a fold of the body wall; the atrial ampulla covered by large clusters of discrete prostatic glands, entering the atrium through narrow passages; vasa deferentia joining the atrium in the basal third of its length, and opening to the atrial lumen at the apical end.

**Table 5.** *Stylodrilus* species with simple-pointed chaetae, elongate to tubular atrium, vas deferens not entering postatrial segment.

Species	Atrium position (segment)	Atrial ampulla/duct size	Prostate	Vas deferens junction to atrium	Penis	Spermatheca	Spermathecal ampulla/duct size	Posterior lateral blood vessels
*Stylodrilus curvithecus* Collado et al., 1993	X–XI	Atrium elongate, ampulla pyriform, length > duct	Diffuse, poorly developed	At the base of the ampulla, but open subapically to atrial lumen	Conical, in a fold of the body wall, with muscular bulb and associated glands	Restricted to segment IX	Ampulla folded, length > duct	Absent
*Stylodrilus glandulosus* Giani & Martínez-Ansemil, 1984	X–XI(XII)	Ampulla elongate, length ≈ duct	Diffuse	At the base of the ampulla, but open subapically to atrial lumen	Conical, in a fold of the body wall, with muscular bulb and associated glands	Restricted to segment IX	Ampulla oval, length < duct	Present (not branching)
*Stylodrilus tschaunensis* Morev, 1982	X	Ampulla pyriform, length>duct	Diffuse	At the base of the ampulla, but open apically to the atrial lumen	Absent	In IX	Sac-shaped ampulla, length > duct	Present, short and slightly branching
*Stylodrilus wahkeenensis* Rodriguez & Coates, 1996	IX	Atrial duct absent	Diffuse	Medially	Absent	Ampulla in VII–VIII (genitalia shifted forward)	Ampulla length > duct (both elongate)	Absent
*Stylodrilus coreyi* sp. n.	X–XI	Ampulla tubular, >> duct	In petiolate clusters	At the base of the ampulla, but open apically to atrial lumen	Short penis	Ampulla in IX–X	Ampulla oval, length < duct	Absent

Among this group of species, *Stylodrilus wahkeenensis* Rodriguez & Coates, 1996 can be distinguished from *Stylodrilus coreyi* not only by the remarkable shape of the chaetae (proximal nodulus, hair-like in dorsal bundles, and enlarged, hook-shaped in ventral bundles of segment II), but also by the position and structure of the atrium (in segment IX, small and covered by a simple, diffuse layer of prostatic cells, with no duct or penis observed). Of the other species in that group, *Stylodrilus glandulosus* Giani & Martínez-Ansemil, 1984 and *Stylodrilus curvithecus* Collado et al., 1993 are separated from congeners by clear apomorphies, such as a muscular, bulbous penial sac with associated glandular complex, and a long atrial duct. *Stylodrilus beattiei* Cook, 1975 and *Stylodrilus tschaunensis* Morev, 1982 also have simple-pointed chaetae and vas deferens not penetrating the postatrial segment, but they are well distinguished from this species group by the distinctly petiolate atrium with oval or pyriform ampulla, short atrial duct, and absence of a penis. Other species of the genus in which the posterior vas deferens does not penetrate the post-atrial segment are *Stylodrilus cernosvitovi* Hrabě, 1950, *Stylodrilus mirandus* (Hrabě, 1982), and *Stylodrilus aclotudi* Kaygorodova & Martin, 2008, but they all have bifid chaetae.

*Stylodrilus* species with simple chaetae and an elongate to tubular atrium with short atrial duct include *Stylodrilus absoloni* (Hrabě, 1970), *Stylodrilus lemani* and *Stylodrilus chukotensis* Sokolskaya, 1975, but in these species, the vas deferens penetrates the post-atrial segment, forming a loop. Another species in this group is *Stylodrilus sulci* (Hrabě, 1934), distinguished from *Stylodrilus coreyi* by the median junction of vasa deferentia to the atrium, the entrance of the posterior vas deferens into the postatrial segment, and the absence of a penis.

In North America, there are only five *Stylodrilus* species known so far, one of which is cosmopolitan (*Stylodrilus heringianus* Claparède, 1862). *Stylodrilus beattiei* (Cook, 1975) was the first Nearctic *Stylodrilus* species described, from a cave in West Virginia. Subsequently, *Stylodrilus sovaliki* (Holmquist, 1976) was described from lakes in Alaska. Later, *Stylodrilus californianus* Rodriguez, 1996 was discovered in subterranean waters in eastern California, and *Stylodrilus wahkeenensis* Rodriguez & Coates, 1996, was described from hyporheic waters and small streams associated with subterranean waters of Oregon and southeastern USA.

The low number of *Stylodrilus* species in North America may be related in part to the tendency of researchers in this area to erect new genera for those taxa with very distinct apomorphies (e.g., *Spelaedrilus* Cook, 1975, *Phagodrilus* McKey-Fender & Fender, 1988, *Tenagodrilus* Eckroth & Brinkhurst, 1996), despite a general arrangement of the reproductive system that fits the *Stylodrilus* pattern. This situation indicates the need for a sound revision of the genus, since some *Stylodrilus* species can in fact be closer to other genera.

#### Ecological remarks.

*Stylodrilus coreyi* sp. n. has been collected from seeps and pools in humic coastal plain streams (Drowning and Pettiford Creeks), most commonly outside of the main channel. These habitats have a temporary flow regime, with seasonal drying during summer months. *Stylodrilus coreyi* was mostly collected in detritus over a layer of fine sand. Both streams have very high water quality (NCDENR 2007, 2011), but pH values are higher in Drowning Creek (usually about 5.5) than in Pettiford Creek (< 4.3). This suggests that *Stylodrilus coreyi* tolerates extremely low pH values, but does not require such conditions. Interestingly, lumbriculids found in this kind of habitat have also congeneric relatives in groundwaters (three of five described *Stylodrilus* species in the Nearctic region are subterranean; see also *Cookidrilus pocosinus* remarks, above).

## Discussion

The new taxa show several characters that are interesting in the context of taxonomy of the family Lumbriculidae, and are worth a more general discussion.

### Spermathecal position and number

In the present paper, we describe three species that differ in number and position of spermathecae. The phylogenetic analysis of the family Lumbriculidae performed by Brinkhurst (1989) suggested that these characters were subject to many reversals and have low value in the phylogeny; however, this result contradicts the central role that number and position of spermathecae have played in the taxonomy of the family. Still, only two recognized lumbriculid genera include species with spermathecae both anterior and posterior to the atrial segments, namely, *Styloscolex* and a single *Lumbriculus* species. *Lumbriculus alexandrovi* Popchenko, 1976, is remarkable in having spermathecae in front of, in, and behind atrial segments. Future phylogenetic analyses based on both morphological and molecular data will provide more light on the importance of these characters and their validity in the classification of lumbriculids.

With respect to the spermathecal pores, it is interesting to note that the most common (and thus probably ancestral) position in aquatic oligochaetes is in the anterior part of the segment, in front of the ventral chaetae or even very close to the anterior septum. This is also the most common position within the family Naididae (sensu Erséus et al. 2008), although in the Tubificinae and Limnodriloidinae, the spermathecal pores are usually located just in front of, or at about the level of the chaetal bundles. There are exceptions where the spermathecae are well behind ventral chaetae, such as *Branchiura sowerbyi*, at present classified in Rhyacodrilinae. Thus, it is remarkable that the position of the pores is behind the ventral chaetae in most lumbriculids. It is also noteworthy that among the lumbriculids, several Nearctic taxa have spermathecal pores in front of the ventral chaetae (see taxonomic remarks in *Cookidrilus pocosinus*), whereas this is extremely rare in Palearctic species (Timm and Popchenko 1978 reported an abnormal spermatheca opening in the anterior part of segment VIII in *Tatriella slovenica* Hrabĕ, 1939).

### Prostatic cells

The organization of prostatic cells into petiolate bundles has been reported before in several lumbriculid genera, but to date this character has not been considered diagnostic for genera. Therefore, species with either diffuse or clustered prostatic cells are found within *Trichodrilus* (see Rodriguez and Giani 1994), and *Stylodrilus* (e.g., *Stylodrilus mirus* Chekanovskaya, 1956, or the North American *Stylodrilus sovaliki* (Holmquist, 1976)). Multicellular prostate glands seem to be present in most North American lumbriculids, e.g., species of *Eclipidrilus*, *Rhynchelmis*, *Eremidrilus*, *Kincaidiana* and *Altmanella* Fend, 2009. Clusters of glandular cells connected to the atrium through a single passage have also been reported in some East Asian lumbriculids (e.g., *Stylodrilus mirus*, *Hrabea ogumai* Yamaguchi, 1936, *Yamaguchia toyensis* Fend & Ohtaka, 2004), as well as in a few European taxa (e.g., *Pseudorhynchelmis paraolchonensis* (Giani & Martínez-Ansemil, 1984), and several *Trichodrilus* species in Rodriguez and Giani 1994). The structure of prostatic glands may be subject to interpretation due to fixation or cell density; however, this character has played an important role in the classification of other higher oligochaete taxa, and does require more attention in the Lumbriculidae.

### Penial sheath

“Cuticular penis sheath” has been a confusing term, since different structures may be fundamentally homologous as presumably derived from ectodermal secretions of the developing penis. Holmquist (1985) discussed the problem with reference to tubificids, and defined different resultant structures, reserving the term “sheath” for a rigid structure that disassociates from the soft tissue (thus the penis is free within it). Other authors use the term “penis sheath” for any cuticular covering. In the Lumbriculidae, this has generally been restricted to the well-defined structure that covers the ectal part of the penis of some species of *Styloscolex* (e.g., Cook 1971, Semernoy 2004), and we have adopted this broader definition.

In *Lumbriculus variegatus*, Hesse (1902) described a tubular cuticular penis, and Holmquist (1976) also referred to the presence of a “slender cuticular penis” in *Lumbriculus inconstans* (Smith, 1895), *Lumbriculus genitosetosus* (Holmquist, 1976) and *Lumbriculus ambiguus* (Holmquist, 1976). Our observations indicate the presence of a soft cuticular layer on the penis of a sectioned *Lumbriculus japonicus* Yamaguchi from Yamaguchi’s collection (Fig. 2O), although it appears to be present in some, but not all, dissected *Lumbriculus* specimens in S. Fend’s collections. In contrast, in *Styloscolex*, a rigid cuticular layer encloses the attenuated epithelial tube of the penis within a non-cuticular penial sac (Fig. 2N), thus resembling penes of *Sylphella* (Fig. 2M). This external cuticular layer should not be confused with the internal cuticular lining described for penes of other lumbriculids, such as *Eclipidrilus frigidus* Eisen (see Fend 2005).

### The importance of the unusual aquatic habitats in systematics and conservation

Biomonitoring programs are well developed for larger streams and rivers (Lenat 1988, 1993, Lenat and Barbour 1994), but evaluation of smaller streams, temporary streams and swamp streams can be more difficult (Lenat 2003). Many biological monitoring systems use the taxa richness of intolerant EPT groups (Ephemeroptera, Plecoptera, Trichoptera) as an important metric, but these groups may be relatively sparse in temporary streams or acid waters. In this situation, it may be more informative to evaluate other macroinvertebrate groups, including oligochaete worms. Studies in North Carolina (Lenat and Fend, in preparation) suggest that Lumbriculidae can be abundant and diverse in temporary streams and swamp streams, such that the identification of lumbriculid species can make an important contribution to both water quality assessments and consideration of conservation value. An inventory of unaltered (reference) sites from all kinds of aquatic habitats is needed to complement information given by more typical riffle sampling, in order to conserve an acceptable level of regional species richness (Curry et al. 2012).

The presence of lumbriculids can be particularly useful when the diversity of the macroinvertebrate community is limited by low pH (Lake Run, Pettiford Creek), lack of water during summer months (Lake Run, Pettiford Creek, Drowning Creek floodplain, UT Pokeberry Creek) or small size (UT Pokeberry Creek). UT Pokeberry Creek presents a very interesting example where the larger creek was severely affected by nonpoint source runoff, but the small seeps (which supported a variety of rare invertebrates) were shown to worthy of environmental protection (Lenat, unpublished data). The study and mapping of unusual aquatic habitats (including pools, seeps, and swamps) will bring interesting novelties to the field of biodiversity and ecology, since the range of environmental conditions and microhabitats differs from those commonly studied in rivers. Future collections from these poorly studied habitats can also give light to the fields of systematics and zoogeography. For example, springs or swamps in southeastern North America constitute the only known habitat for three recently-described, monotypic lumbriculid genera (*Sylphella*, *Pilaridrilus*, *Pararhynchelmis*), and have also provided dramatic range extensions for such genera as *Rhynchelmis*, *Cookidrilus*, and *Altmanella*.

## Supplementary Material

XML Treatment for Sylphella

XML Treatment for Sylphella puccoon

XML Treatment for Cookidrilus pocosinus

XML Treatment for Stylodrilus coreyi

## Appendix 1

**Key of the known North American *Stylodrilus* species**

**Table:**

1	Chaetae bifid, with short upper tooth (some chaetae in anterior segments can be simple-pointed)	**2**
–	All chaetae simple-pointed	**3**
2	Penis long, permanently protruded, atrium oval, spermathecal duct long	***Stylodrilus heringianus* Claparède, 1862**
	Medium size worms (0.6–1 mm diameter). Prostatic cells forming clusters, thick (to 30 μm) vasa deferentia join atrial ampulla apically, posterior vas deferens penetrates the postatrial segment. 2 pairs short, unbranched lateral blood vessels in posterior segments. Widespread in Northern USA and Canada, including the Great Lakes.	
–	Penis short, atrium elongate, spermathecal duct very short	***Stylodrilus californianus* Rodriguez, 1996**
	Small worms (0.2–0.5 mm diameter). Vasa deferentia join the atral ampulla medially and open to large lumen apically after running through the atrial musculature, posterior vas deferens penetrates the postatrial segment. Ridgecrest, eastern California, phreatic waters (in a well).	
3	Chaetae with markedly proximal nodulus, dorsals with very long (hair-like) distal end	***Stylodrilus wahkeenensis* Rodriguez & Coates, 1996**
	Small worms (0.3–0.4 mm diameter). Atria in IX, tubular, posterior vas deferens not penetrating the postatrial segment, atrial duct and penis absent, spermathecal duct long and thick. Oregon, Alabama and Tennessee; hyporheic and small rivers associated with subterranean waters.	
–	Chaetae with distal nodulus	**4**
4	Atrium elongate, duct weakly separated from ampulla and of similar diameter	***Stylodrilus coreyi* sp. n.**
	Small worm (0.3–0.4 mm diameter). Posterior vas deferens not penetrating the postatrial segment. North Carolina, pocosin, acidic waters.	
–	Atrium pedunculate, duct clearly separated from ampulla, and much narrower	**5**
5	Posterior vas deferens does not enter the postatrial segment; vasa deferentia join atrial ampulla and open to the lumen basally	***Stylodrilus beattiei* Cook, 1975**
	Medium size worm (0.7–0.9 mm diameter). Prostatic cells small and disappearing soon after mating; lateral blood vessels absent in posterior segments. Tub Cave, West Virginia.	
–	Posterior vas deferens penetrates the postatrial segment; vasa deferentia join atrial ampulla basally and open to the lumen medially	***Stylodrilus sovaliki* (Holmquist, 1976)**
	Medium to large size (about 1 mm diameter). Prostatic cells in bundles, posterior lateral blood vessels branched. Alaska, rivers.	

## Appendix 2

**Appendix 2:** Location of study sites (all in North Carolina, USA), and species described in present paper.

Stream	Latitude	Longitude	Ecoregion	Species described
Unnamed tributary to Pokeberry Cr	N35.8267	W79.1013	Piedmont	*Sylphella puccoon* gen. n., sp. n
Pettiford Cr	N34.7471	W77.0221	Coastal Plain	*Cookidrilus pocosinus* sp. n. *Stylodrilus coreyi* sp. n.
Lake Run	N34.7773	W78.6646	Coastal Plain	*Cookidrilus pocosinus* sp. n.
Anderson Cr tributary to the Lower Little River	N35.2661	W78.8192	Sandhills	*Cookidrilus pocosinus* sp. n.
Drowning Cr	N35.0662	W79.5496	Sandhills	*Cookidrilus pocosinus* sp. n. *Stylodrilus coreyi* sp. n.
